# Supplementary material for: Staphylococcus aureus counters organic acid anion-mediated inhibition of peptidoglycan cross-linking through robust alanine racemase activity
Source: bioRxiv. 2024 Jan 16:2024.01.15.575639. Preprint. [Version 1] doi: 10.1101/2024.01.15.575639 (PMC10827132; doi:10.1101/2024.01.15.575639)
Supplement: Supplementary file 1 [file NIHPP2024.01.15.575639v1-supplement-1.pdf]

bioRxiv preprint doi: https://doi.org/10.1101/2024.01.15.575639; this version posted January 17, 2024. The copyright holder for this preprint (which was not certified by peer review) is the author/funder, who has granted bioRxiv a license to display the preprint in perpetuity. It is made available under aCC-BY 4.0 International license.

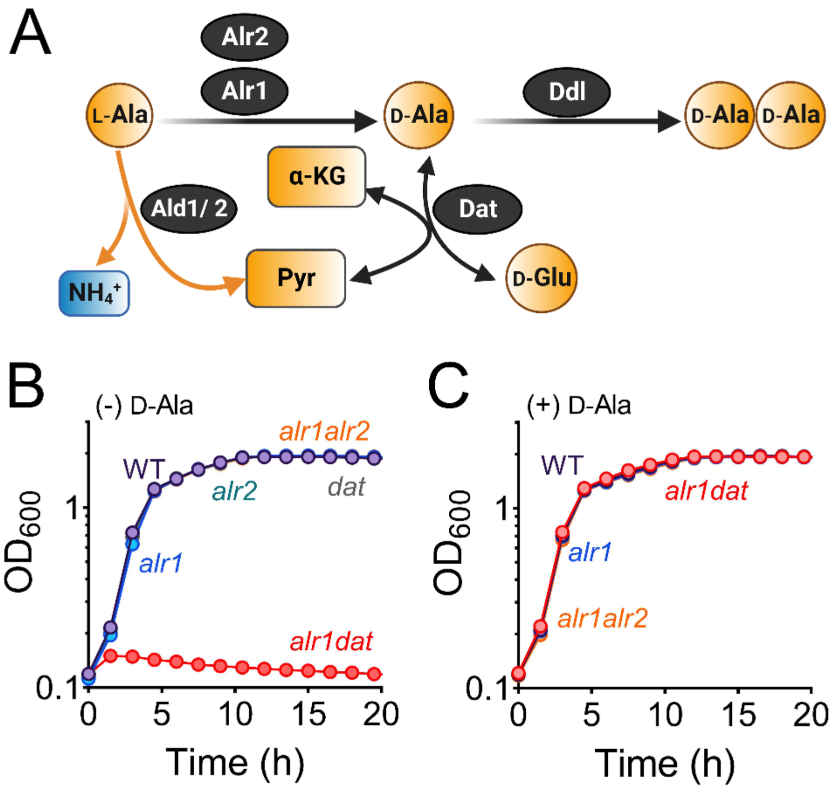

Figure 1-figure supplement 1. Alr1 and Dat are the primary routes of D-Ala production in *S. aureus*. **(A)** Schematic of the predicted D-Ala-D-Ala generating pathways in *S. aureus*. Growth curves of various *S. aureus* strains grown in the **(B)** absence or **(C)** presence of 5 mM D-Ala (n=3, mean ± SD).

bioRxiv preprint doi: https://doi.org/10.1101/2024.01.15.575639; this version posted January 17, 2024. The copyright holder for this preprint (which was not certified by peer review) is the author/funder, who has granted bioRxiv a license to display the preprint in perpetuity. It is made available under aCC-BY 4.0 International license.

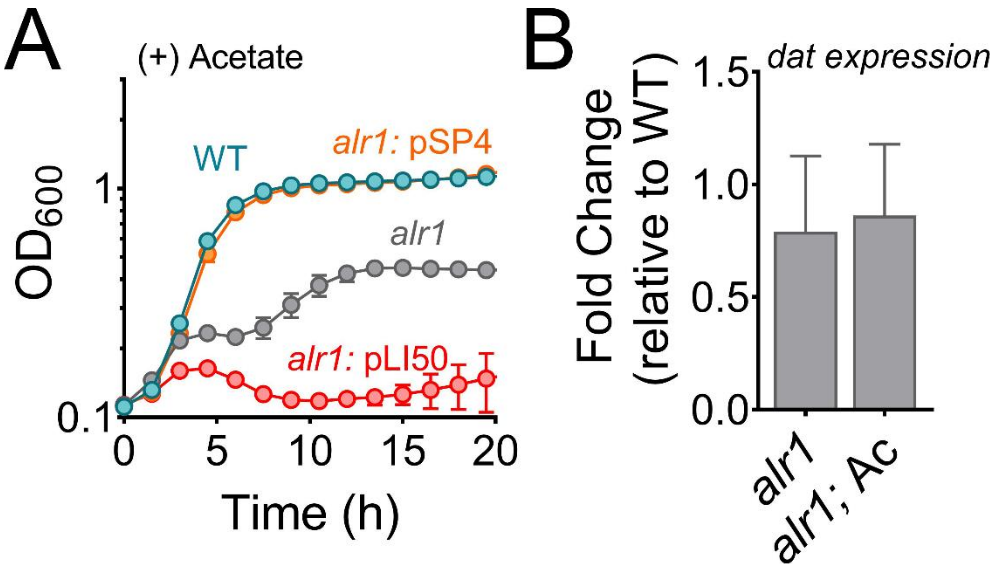

Figure 2-figure supplement 1. Overexpression of *dat* rescues the growth defect of the *alr1* mutant (A) *dat* was cloned in a multicopy vector (pSP4) controlled by its native promoter. The *S. aureus* strains containing pSP4 and pLI50 (empty vector) were grown in TSB supplemented with 20 mM acetate **(B)** RT-qPCR analysis of *dat* expression in the *alr1* mutant in the presence or absence of 20 mM acetic acid (n=3, mean ± SD).

bioRxiv preprint doi: https://doi.org/10.1101/2024.01.15.575639; this version posted January 17, 2024. The copyright holder for this preprint (which was not certified by peer review) is the author/funder, who has granted bioRxiv a license to display the preprint in perpetuity. It is made available under aCC-BY 4.0 International license.

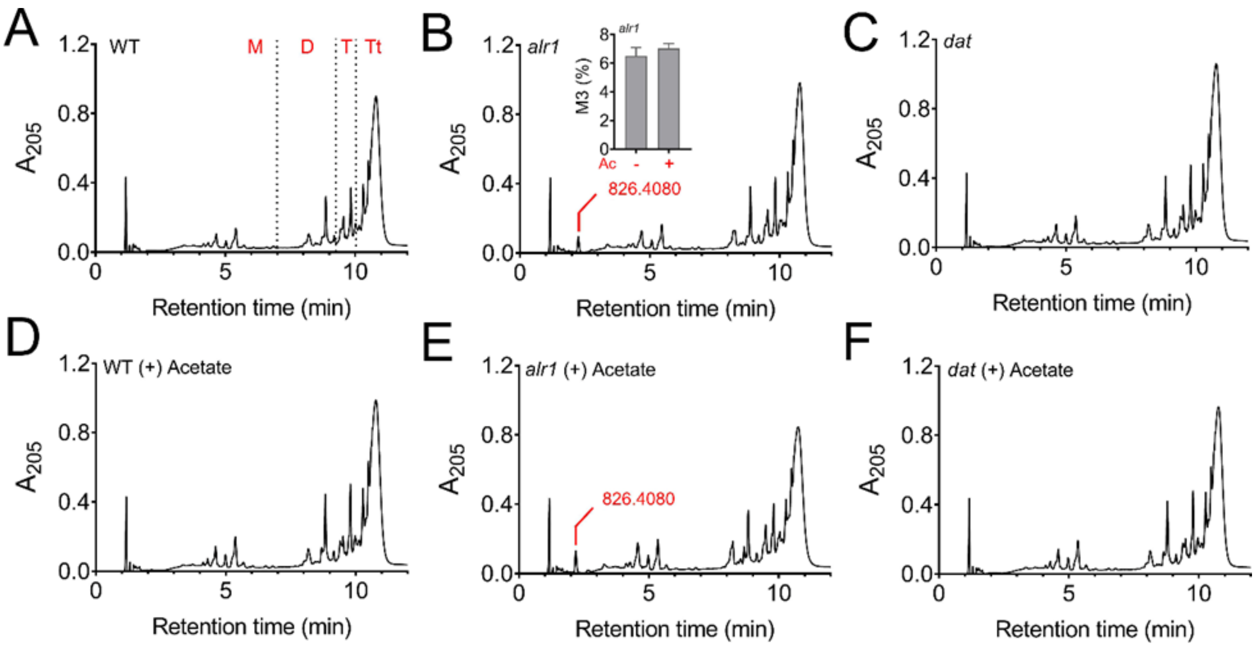

Figure 4-figure supplement 1. Muropeptide analysis. Representative **c**hromatograms of muropeptide extracts from **(A-C)** WT, *alr1* and *dat* mutants, and following **(D-F)** acetate intoxication. A unique peak corresponding to NAG-NAM-AEK (M3, m/z, Da: 826.4080) was identified in the *alr1* mutant. The peak area of M3 was normalized to the total area of peaks observed in the chromatogram and expressed as percent (see inset figure in **B**, n=3, mean ± SD). M, monomer; D, dimer; T, trimer, Tt, tetramer.

bioRxiv preprint doi: https://doi.org/10.1101/2024.01.15.575639; this version posted January 17, 2024. The copyright holder for this preprint (which was not certified by peer review) is the author/funder, who has granted bioRxiv a license to display the preprint in perpetuity. It is made available under aCC-BY 4.0 International license.

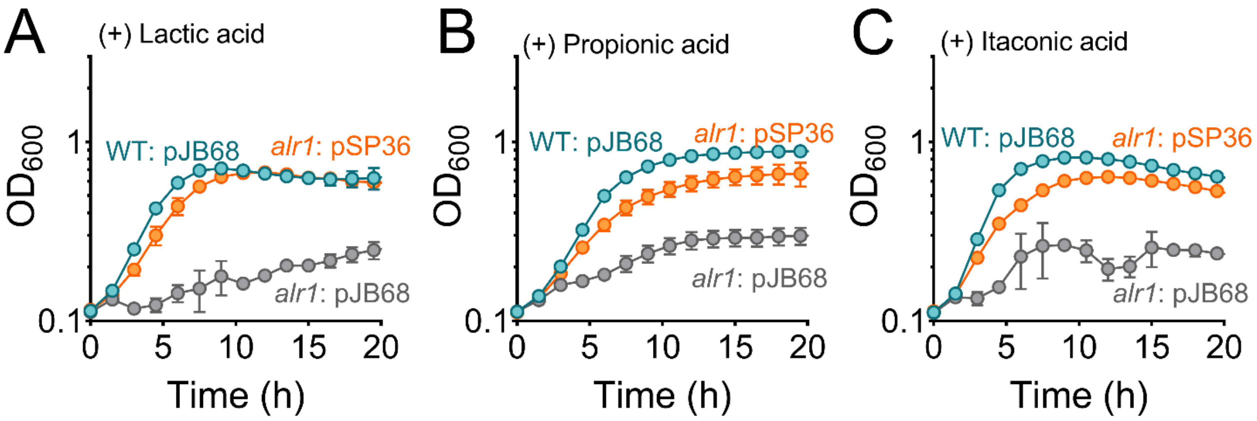

Figure 6-figure supplement 1. Overexpression of *ddl* rescues the growth defect of the *alr1* mutant The growth (OD_{600}) of the WT and *alr1* mutants overexpressing Ddl (pSP36; cadmium inducible expression of *ddl*) in TSB supplemented with **(A)** lactic acid (40 mM), **(B)** propionic acid (20 mM) and **(C)** Itaconic acid (20 mM) in the presence or absence of 5 mM D-Ala (n=3, mean ± SD).

bioRxiv preprint doi: https://doi.org/10.1101/2024.01.15.575639; this version posted January 17, 2024. The copyright holder for this preprint (which was not certified by peer review) is the author/funder, who has granted bioRxiv a license to display the preprint in perpetuity. It is made available under aCC-BY 4.0 International license.

Table S1: Effect of acetate on Ddl activity

Substrate	Condition	K_m (mM)	V_{max} (μM min^{-1})	k_{cat} (min^{-1})
D-**Ala**	0 mM Acetate	7.0 ± 0.6	16.0 ± 0.4	80.1 ± 2.1
	100 mM Acetate	6.8 ± 0.6	10.9 ± 0.3	54.4 ± 1.6
	300 mM Acetate	8.4 ± 0.6	6.3 ± 0.1	31.5 ± 0.7
**ATP**	0 mM Acetate	0.6 ± 0.1	12.3 ± 1.1	61.7 ± 5.7
	200 mM Acetate	0.8 ± 0.2	6.9 ± 0.8	34.4 ± 3.8
	300 mM Acetate	0.8 ± 0.3	4.0 ± 0.7	19.9 ± 3.6

bioRxiv preprint doi: https://doi.org/10.1101/2024.01.15.575639; this version posted January 17, 2024. The copyright holder for this preprint (which was not certified by peer review) is the author/funder, who has granted bioRxiv a license to display the preprint in perpetuity. It is made available under aCC-BY 4.0 International license.

Table S2: Impact of acetate on Ddl stability assessed by DSF

Sample	Tm D (ºC)	ΔTm D (ºC)*
Ddl	45.0 ± 0.0	-
Ddl + Sodium acetate (Ac)	48.7 ± 0.0	3.7
Ddl + ATP	46.4 ± 0.0	1.4
Ddl + ATP + Ac	48.9 ± 0.1	3.9
Ddl + ADP	41.9 ± 0.1	-3.2
Ddl + ADP + Ac	49.7 ± 0.1	4.0
Ddl + D-Ala	49.2 ± 0.1	4.2
Ddl + D-Ala + Ac	49.6 ± 0.0	4.5
Ddl + D-Ala + ATP + Ac	47.1 ± 0.1	2.0
Ddl + D-Ala + ADP + Ac	49.8 ± 0.0	4.8

*The ΔTm D values are calculated as the difference in melting temperature of the Ddl apo protein to Ddl with added substrates or acetate inhibitor.

bioRxiv preprint doi: https://doi.org/10.1101/2024.01.15.575639; this version posted January 17, 2024. The copyright holder for this preprint (which was not certified by peer review) is the author/funder, who has granted bioRxiv a license to display the preprint in perpetuity. It is made available under aCC-BY 4.0 International license.

Table S3: Refinement statistics of DdI/Acetate structure

Resolution Range	48.21 – 1.92 (1.989 – 1.92)
Space group	P 2 21 21
Unit cell	55.123 65.817 99.423 90 90 90
Total reflections	173526 (12202)
Unique reflections	20366 (2806)
Multiplicity	8.5 (9.3)
Completeness (%)	94.80 (50.46)
Mean I/sigma(I)	10.99 (1.72)
Wilson B-factor	37.29
R-merge	0.097 (0.33)
R-meas	0.104 (0.35)
R-pim	0.036 (0.12)
CC1/2	0.998 (0.95)
CC*	0.999 (0.99)
Reflections used in refinement	26870 (1416)
Reflections used for R-free	1601 (92)
R-work	0.21 (0.42)
R-free	0.27 (0.47)
CC (work)	0.223 (0.07)
CC (free)	0.217 (-0.16)
Number of non-hydrogen atoms	2963
macromolecules	2777
ligands	10
solvent	176
Protein residues	355
RMS(bonds)	0.009
RMS(angles)	1.10
Ramachandran favored (%)	93.70
Ramachandran allowed (%)	6.02
Ramachandran outliers (%)	0.29
Rotamer outliers (%)	0.00
Clashscore	6.88
Average B-factor	43.60

bioRxiv preprint doi: https://doi.org/10.1101/2024.01.15.575639; this version posted January 17, 2024. The copyright holder for this preprint (which was not certified by peer review) is the author/funder, who has granted bioRxiv a license to display the preprint in perpetuity. It is made available under aCC-BY 4.0 International license.

macromolecules	43.60
ligands	45.09
solvent	43.64

bioRxiv preprint doi: https://doi.org/10.1101/2024.01.15.575639; this version posted January 17, 2024. The copyright holder for this preprint (which was not certified by peer review) is the author/funder, who has granted bioRxiv a license to display the preprint in perpetuity. It is made available under aCC-BY 4.0 International license.

Table S4: Glide scores from molecular docking studies of organic anions

Organic Anion	ATP binding site	D-Ala binding site
L-lactate	-4.851	-4.431
Propionate	-2.317	-1.883
Itaconate	-3.572	-3.575

bioRxiv preprint doi: https://doi.org/10.1101/2024.01.15.575639; this version posted January 17, 2024. The copyright holder for this preprint (which was not certified by peer review) is the author/funder, who has granted bioRxiv a license to display the preprint in perpetuity. It is made available under aCC-BY 4.0 International license.

Table S5: Strains used in this study

Strains	Description	Source
E. coli Electro-Ten-Blue	General plasmid maintenance strain	Stratagene
S. aureus RN4220	Restriction-deficient strain is routinely used as a transformation intermediate	(54)
S. aureus RN4220: pRN7023	Restriction deficient strain carrying pRN7023 plasmid containing integrase gene routinely used as a transformation intermediate	(39)
E. coli DH5α	General plasmid maintenance strain	Thermo Fisher
E. coli BL21(DE3)	Protein over-expression and purification strain	Novagen
S. aureus JE2	*S. aureus* USA300 LAC cured of all 3 native plasmids	(37)
JE2 *alr1*	*bursa aurealis* transposon mutant, Erm^R	NTML
JE2 *alr1::alr1*	WT copy of *alr1* complemented at the SaPI1 site of *bursa aurealis* transposon mutant, Erm^R	This study
JE2 *citZ*	*bursa aurealis* transposon mutant, Erm^R	NTML
JE2 *citZalr1*	*bursa aurealis* transposon mutant, Erm^R, Kan^R	This study
JE2 *dat*	*bursa aurealis* transposon mutant, Erm^R	NTML
JE2 Δ*alr2*	Inframe isogenic deletion mutant of JE2	This study
JE2 *alr1*Δ*alr2*	*bursa aurealis* transposon mutant, Erm^R, transduced into inframe isogenic deletion mutant JE2 Δ*alr2*	This study
JE2 *alr1dat*	*bursa aurealis* transposon mutant, Erm^R, Tet^R	This study
JE2 alr1: *dat*	pLI50 *dat* plasmid (pSP4) transduced into *bursa aurealis* transposon mutant, Erm^R	
JE2 *pepV*$^{\Delta SD1\text{-}467}$	Isogenic deletion mutant of SD1 and *pepV*	This study
JE2 *alr1pepV*$^{\Delta SD1\text{-}467}$	*bursa aurealis* transposon mutant, Erm^R transduced into isogenic deletion mutant of SD1 and *pepV*	This study
JE2 Δ*pepV*	Inframe isogenic deletion mutant of JE2	This study
JE2 *alr1*Δ*pepV*	*bursa aurealis* transposon mutant, Erm^R transduced into inframe isogenic deletion mutant JE2 Δ*pepV*	This study
JE2 *pepV*^{Q12STOP}	Glutamine to STOP codon substitution at 12th amino acid position in PepV	This study
JE2 *alr1pepV*^{Q12STOP}	*Bursa aurealis* transposon mutant, Erm^R transduced into glutamine to STOP codon substitution at 12th amino acid position in PepV	This study
JE2 WT: pJB68	pJB68 transduced into WT JE2	This study
JE2 WT: pSP36	pSP36 transduced into WT JE2	This study
JE2 *alr1*: pJB68	pJB68 transduced into JE2 *alr1*	This study
JE2 *alr1*: pSP36	pSP36 transduced into WT *alr1*	This study
JE2 WT::pAQ59	pAQ59 empty vector inserted at the SaPI1 site of WT JE2	This study
JE2 WT::pAS8	*dat* (under its native promoter) inserted at the SaPI1 site of WT JE2	This study

bioRxiv preprint doi: https://doi.org/10.1101/2024.01.15.575639; this version posted January 17, 2024. The copyright holder for this preprint (which was not certified by peer review) is the author/funder, who has granted bioRxiv a license to display the preprint in perpetuity. It is made available under aCC-BY 4.0 International license.

Table S6: Plasmids used in this study

Plasmids	Description	Source
pLI50	*E. coli*- *S. aureus* shuttle vector	(55)
pJB38	*E. coli*- *S. aureus* allelic exchange vector	(38)
pJC1111	*E. coli*- *S. aureus* SaPI1 site integration vector	(39)
pJB68	*E. coli*- *S. aureus* cadmium inducible shuttle vector	(38)
pET28a	Expression vector for purification of protein in *E. coli* BL21 (DE3)	Novagen
pAS3	pJC1111 based vector for integration of WT copy of *alr1* at the SaPI1 site	This study
pAS2	pJB38 based vector for *alr2* chromosomal deletion	This study
pSP4	pLI50 *dat* (under control of its native promoter)	This study
pSP19	pJB38 based vector for *pepV* SD1 chromosomal deletion	This study
pSP20	pJB38 based vector for *pepV* SD1-467 chromosomal deletion	This study
pSP16	pJB38 based vector for *pepV* chromosomal deletion	This study
pSP15	pJB38 based vector for substitution of chromosomal *pepV* with *pepV*^{Q12STOP}	This study
pSP36	pJB68 based vector for overexpression of *ddl*	This study
pSP32	pET28a based vector for purification of full length Ddl (C-terminal his tag)	This study
pAQ59	*E. coli*- *S. aureus* SaPI1 site integration vector with pSC101 ori region	(56)
pAS8	pAQ59 based vector for integration of *dat* gene under its native promoter at the SaPI1 site	This study

bioRxiv preprint doi: https://doi.org/10.1101/2024.01.15.575639; this version posted January 17, 2024. The copyright holder for this preprint (which was not certified by peer review) is the author/funder, who has granted bioRxiv a license to display the preprint in perpetuity. It is made available under aCC-BY 4.0 International license.

Table S7: Primers used in this study

**Gene/Modification**	**Primer name**	**Primer sequence (5' – 3')**
alr1	alr1_F	TGCTGACGAACCAGGAGATA
	alr1_R	TGTAGTTGGGTCAGTAGCTG
alr1 complementation	alr1_comp_F	CGGCCGCTGCATGCCTGCAGACATGAGCAACGTAAAATTG
	alr1_comp_R	AGCTCGGTACCCGGGGATCCAATGACCTTTAATTACTCTAATGATAAC
citZ	1641_F	CAGCGGAGACTAAAATAAGTTC
	1641_R	CCCAATCTCAGATAACATCGTC
dat	dat_F	ACTATAGGTGGCGGTACTTA
	dat_R	ACCATCGGATATCTTCAACG
alr2 deletion	alr2_UP_F	CGAGGCCCTTTCGTCTTCAATACTTAGAAGGTAATGGCTC
	alr2_UP2_R	TCATAGCACTTGCTGTCAATGTATTACAC
	alr2_DN2_F	ATTGACAGCAAGTGCTATGAATCATGATTC
	alr2_DN_R	TTGCATGCCTGCAGGTCGACGCTTCTTCATTTCTATTAACAAG
dat complementation	dat promoter_F	CCTTTCGTCTTCAAGAATTCGATGTGAGTAGGACAGAAATG
	dat promoter_R	TTTTTTCCATTCGAAATCGACTTCCTTTTTTC
	dat_pLI50_F	TCGATTTCGAATGGAAAAAATTTTTTTAAATGGTG
	dat_pLI50_R	TTGCATGCCTGCAGGTCGACCGAAAGTTGATAAATTTAAGTAATTTAATC

bioRxiv preprint doi: https://doi.org/10.1101/2024.01.15.575639; this version posted January 17, 2024. The copyright holder for this preprint (which was not certified by peer review) is the author/funder, who has granted bioRxiv a license to display the preprint in perpetuity. It is made available under aCC-BY 4.0 International license.

TSS identification of *dat* operon	pepV_TSS_R1	P-CCATCTCTATGTGCAATTTC
	pepV_TSS_R2	GCGTCTTCTGATGCTTTTGC
	pepV_TSS_F3	GTCCTCGTAAGGCATTAGAC
	M13F (-20)	GTAAAACGACGGCCAG
	M13R	CAGGAAACAGCTATGAC
*pepV*ΔSD1-467	RBS1pepV_UP_F	CCTTTCGTCTTCAAGAATTCAGCGACGCAATTAGGAAC
	RBS1pepV_UP_R	TTATTCCTCCTTTTTCTATAAGTTAAATTCTATTTTACATGAAAAG
	RBS1pepV_DN_F	TATAGAAAAAGGAGGAATAATATATGGAAAAAATTTTTTTAAATG
	RBS1pepV_DN_R	TATAGAAAAAGGAGGAATAATATATGGAAAAAATTTTTTTAAATG
pepV deletion	pepV_UP2_F	CCTTTCGTCTTCAAGAATTCAACAATTAAAGAAGTAAAAACAAATC
	pepV_UP2_R	TTTTTTCCATTCGAAATCGACTTCCTTTTTTC
	pepV_DN2_F	TCGATTTCGAATGGAAAAAATTTTTTTAAATGGTG
	pepV_DN2_R	TTGCATGCCTGCAGGTCGACTTTCAACTGAAAATGAGAAAC
*pepV*Q12STOP	pepV_STOP_UP_F	CCTTTCGTCTTCAAGAATTCCAAATCCGAAAGAATATGC
	pepV_STOP_UP_R	TAATGATTTAATCTTCGTATTGTTGAACTTTTTC
	pepV_STOP_DN_F	ATACGAAGATTAAATCATTAATGACTTAAAAGGATTATTAG
	pepV_STOP_DN_R	TTGCATGCCTGCAGGTCGACAAAGACCTGCGTTTTCATTATC
ddl overexpression plasmid	ddl_pJB68_F	TTTATAAGGAGGAAAAACATATGACAAAAGAAAATATTTGTATCG

bioRxiv preprint doi: https://doi.org/10.1101/2024.01.15.575639; this version posted January 17, 2024. The copyright holder for this preprint (which was not certified by peer review) is the author/funder, who has granted bioRxiv a license to display the preprint in perpetuity. It is made available under aCC-BY 4.0 International license.

	ddl_pJB68_R	GAATAGGCGCGCCTGAATTCATCCATGATTGAATTTGCTTTAATG
Ddl purification plasmid	ddl_C_Histag_F	CTTTAAGAAGGAGATATACCATGACAAAAGAAAATATTTGTATCG
	ddl_C_Histag_R	CAGTGGTGGTGGTGGTGGTGGTCAATTTTGTATTTATTTTTCTGTTTATC
ddl RT-qPCR	ddl_RT_F	GGGCTTTTTGAAGTTTTGGA
	ddl_RT_R	TGGTAACCCTCGATGTTCAA
murF RT-qPCR	murF_RT_F	TCACAATTGATTCACGAGCA
	murF_RT_R	CCCAGCACCATCTTGTAATG
dat RT-qPCR	dat RT_F	GATGGTTACGTTGCGACATT
	dat RT_R	CACCTCGATGTTGAATTGCT
sigA RT-qPCR	JE2_RT_sigA_F	AACTGAATCCAAGTGATCTTAGTG
	JE2_RT_sigA_R	TCATCACCTTGTTCAATACGTTTG
dat insertion at the SaPI1 site (pAS8)	dat_UP2_F	GAGCCGCTGCATGCCTGCAGGATGTGAGTAGGACAGAAATG
	dat_UP_R	TTTTTTCCATTCGAAATCGACTTCCTTTTTTC
	dat_DN_F	TCGATTTCGAATGGAAAAAATTTTTTTAAATGGTG
	dat_DN_R	AGCTCGGTACCCGGGGATCCCGAAAGTTGATAAATTTAAGTAATTTAATC

bioRxiv preprint doi: https://doi.org/10.1101/2024.01.15.575639; this version posted January 17, 2024. The copyright holder for this preprint (which was not certified by peer review) is the author/funder, who has granted bioRxiv a license to display the preprint in perpetuity. It is made available under aCC-BY 4.0 International license.

Table S8: Table of Multiple Reaction Monitoring (MRM) transitions

Metabolite	Polarity	MRM (Q1/Q3)	CE (V)	DP (V)	RT	Column
L-Ala	(+)	90.1 / 44.0	17	65	6.4	C
D-Ala	(+)	90.1 / 44.0	17	65	9.5	C
D-Ala-D-Ala	(+)	161.0 / 44.2	30.5	65	3.4	XB
UDP-NAG	(-)	606.0 / 79.0	-149	-80	12.7	XB
UDP-NAM	(-)	678.1 / 79.0	-120	-80	13.2	XB
UDP-NAM-A	(-)	749.1 / 403.0	-42	-90	13.4	XB
UDP-NAM-AE	(-)	878.2 / 403.0	-48	-105	14.3	XB
UDP-NAM-AEK	(-)	1006.2 / 403.0	-50	-130	14.9	XB
UDP-NAM-AEKAA	(-)	1148.5 / 403.0	-55	-140	14.6	XB
NAM	(-)	292.0 / 89.0	-16	-30	6.2	XB
NAG	(+)	204.0 / 138.1	18.9	30	5.6	XB
Br-ATP (IS)	(-)	588.0 / 159.0	-38.9	-60	6.0	XB
Ribitol (IS)	(-)	151.1 / 89.0	-14.8	-60	5.3	XB

CE: Collision energy ;DP: Declustering potential; RT: Retention Time; C: CHIROBIOTIC® T column; XB: XBridge Amide column

bioRxiv preprint doi: https://doi.org/10.1101/2024.01.15.575639; this version posted January 17, 2024. The copyright holder for this preprint (which was not certified by peer review) is the author/funder, who has granted bioRxiv a license to display the preprint in perpetuity. It is made available under aCC-BY 4.0 International license.

Table S9: HRMS base peak identification of isotopologues

	Metabolite	Isotopologues($^{13}C^{15}N$)	Base peak (m/z)
**D-Ala-D-Ala (Positive mode)**			
1	C6H12N2O3	C_0N_0	161.0921
2	[13]C1C5H12N2O3	C_1N_0	162.0954
3	[13]C2C4H12N2O3	C_2N_0	163.0988
4	[13]C3C3H12N2O3	C_3N_0	164.1021
5	[13]C3C3H12[15]N1N1O3	C_3N_1	165.09917
6	[13]C3C3H12[15]N2O3	C_3N_2	166.0962
7	[13]C4C2H12N2O3	C_4N_0	165.10549
8	[13]C5C1H12N2O3	C_5N_0	166.10884
9	[13]C6H12N2O3	C_6N_0	167.1122
10	[13]C6H12[15]N1N1O3	C_6N_1	168.1092
11	[13]C6H12[15]N2O3	C_6N_2	169.1063
12	[13]C1C5H12[15]N1N1O3	C_1N_1	163.09246
13	[13]C1C5H12[15]N2O3	C_1N_2	164.0895
14	[13]C2C4H12[15]N1N1O3	C_2N_1	164.0958
15	[13]C2C4H12[15]N2O3	C_2N_2	165.09285
16	[13]C4C2H12[15]N1N1O3	C_4N_1	166.1025
17	[13]C4C2H12[15]N2O3	C_4N_2	167.09956
18	[13]C5C1H12[15]N1N1O3	C_5N_1	167.10588
19	[13]C5C1H12[15]N2O3	C_5N_2	168.1029
20	C6H12[15]N1N1O3	C_0N_1	162.0891
21	C6H12[15]N2O3	C_0N_2	163.0861
**D-Glu (Negative mode)**			
1	C5H9NO4	C_0N_0	146.04588
2	C5H9[15]NO4	C_0N_1	147.04292
3	[13]C1C4H9NO4	C_1N_0	147.04924
4	[13]C2C3H9NO4	C_2N_0	148.05259
5	[13]C2C3H9[15]NO4	C_2N_1	149.04963
6	[13]C1C4H9[15]NO4	C_1N_1	148.04627
7	[13]C3C2H9[15]NO4	C_3N_1	150.05298
8	[13]C4C1H9[15]NO4	C_4N_1	151.05634
9	[13]C5H9[15]NO4	C_5N_1	152.05969
10	[13]C3C2H9NO4	C_3N_0	149.05595
11	[13]C4C1H9NO4	C_4N_0	150.0593
12	[13]C5H9NO4	C_5N_0	151.06266
